# Promotion of bone formation and antibacterial properties of titanium coated with porous Si/Ag-doped titanium dioxide

**DOI:** 10.3389/fbioe.2022.1001514

**Published:** 2022-10-21

**Authors:** Quanming Zhao, Jieshi Wu, Yankun Li, Ruisheng Xu, Xingyuan Zhu, Yang Jiao, Rui Luo, Xiaohui Ni

**Affiliations:** ^1^ Department of Orthopedics, Guizhou Provincial People’s Hospital, Guiyang, Guizhou, China; ^2^ Department of Orthopaedics, Affiliated Hospital of Jiangnan University, Wuxi, Jiangsu, China; ^3^ Department of Orthopedics, Dafeng People’s Hospital, Yancheng, Jiangsu, China; ^4^ Department of Stomatology, The 7th Medical Center, Chinese PLA General Hospital, Beijing, China

**Keywords:** titanium, plasma oxidation, porous Si/Ag-loaded TiO_2_ coating, osteogenic property, antibacterial property

## Abstract

Implant materials are mainly used to repair and replace defects in human hard tissue (bones and teeth). Titanium (Ti) and Ti alloys are widely used as implant materials because of their good mechanical properties and biocompatibilities, but they do not have the ability to induce new bone formation and have no antibacterial properties. Through surface modification, Ti and its alloys have certain osteogenic and antibacterial properties such that Ti implants can meet clinical needs and ensure integration between Ti implants and bone tissue, and this is currently an active research area. In this study, bioactive Si and Ag were introduced onto a Ti surface by plasma oxidation. The surface morphology, structure, elemental composition and valence, surface roughness, hydrophilicity and other physical and chemical properties of the coating were characterized by scanning electron microscopy (SEM), X-ray diffraction (XRD), X-ray photoelectron spectroscopy (XPS), a profiler and a contact angle meter (CA). Adhesion and extensions of osteoblasts on the surface of the material were observed by scanning electron microscopy, and mineralization of osteoblasts on the surface of the material were observed by alizarin red staining. The antibacterial properties of the material were tested by culturing *Staphylococcus aureus* on the surface of the material. The osteogenic properties of Ti implants with porous Si/Ag TiO_2_ (TCP-SA) coatings were evaluated with *in vivo* experiments in rats. The results showed that Si and Ag were successfully introduced onto the Ti surface by plasma oxidation, and doping with Si and Ag did not change the surface morphology of the coating. The osteoblasts showed good adhesion and extension on the surfaces of Si/Ag coated samples, and the porous Si/Ag TiO_2_ coating promoted cell proliferation and mineralization. The bacterial experiments showed that the porous TiO_2_ coatings containing Si/Ag had certain antibacterial properties. The animal experiments showed that Si/Ag-coated Ti implants promoted integration between the implants and the surrounding bone. It was concluded that the porous Si/Ag TiO_2_ coating on the Ti surface had good osteogenic and antibacterial properties and provides an optimal strategy for improving the osteogenic and antibacterial properties of Ti implants.

## 1 Introduction

Titanium and its alloys have been widely used in spinal interbody fusion cages, artificial joints, dental implants and as replacement and repair materials for other hard tissues. However, Ti is a bio-inert material with low biological activity. Its combinations with bone involve mechanical locking rather than bone binding. It is easy to form a fibrous layer lacking blood vessels at interfaces between the implant and bone tissues, resulting in a series of complications, such as implant infection and prosthesis loosening (Kaur and Singh, 2019). At the same time, Ti has no antibacterial ability, which easily enables bacterial adhesion and implant infection and results in implant failure. After Ti is implanted into the body, it mainly interacts with the implant surface and the surrounding tissue environment. Therefore, modifying the surfaces of Ti implants to solve the problems of implant infection and prosthesis loosening is also an important area in the field of Ti implants.

Plasma oxidation, also known as microarc oxidation, is a surface modification technique for metal materials. This method is a simple process and exhibits high preparation efficiency. This technology is often used on magnesium, aluminum, Ti and their alloys. It causes the surface of the substrate to grow a layer of ceramic film *in situ* (Xue et al., 2020). The coating prepared by plasma oxidation is generally at the micro/nanoscale, which has been proven to be conducive to cell adhesion and proliferation. Plasma oxidation generates microporous coatings by surface modification and also introduces ions with excellent bioactivities and biocompatibilities, such as Ca, P, Mg, Sr, and Cu plasma, into the microporous coatings through electrolytes (Shimabukuro, 2020; Zhang R. et al., 2021; Shen et al., 2022). A previous study found that in an electrolyte containing Ca and P salts, the Ca and P elements of the TiO_2_ coating prepared by plasma oxidation were incorporated into the coating during preparation. *In vivo* research found that a coating containing Ca and P significantly promoted proliferation and differentiation of osteoblasts (Tardelli et al., 2021), so this method is an effective method for improving the biological activity of a Ti surface. In addition, other studies have shown that plasma oxidation improves the roughness of Ti surfaces, thereby promoting osteogenic differentiation of cells (Pan et al., 2019). Therefore, plasma oxidation can promote bone integration with Ti implants.

Silicon (Si) is one of the essential trace elements in the human body and plays an important role in the early stages of bone formation. Bioceramics containing Si, such as Bioglass, Ca-silicate, and wollastonite, are used in the clinic because of their excellent biological activities and biocompatibilities (Rau et al., 2015; Zhao et al., 2020a). The mechanism by which Si-containing ceramics promote the biological activity of a material mainly operates when Si-containing materials are immersed in simulated body fluid. Through ion exchange, the surfaces of materials can effect accumulation of Ca and P ions in the simulated body fluid, thus promoting proliferation and differentiation of osteoblasts on the surface (Ni and Chang, 2009). Other studies have shown that Si-containing biomaterials promote osteoblast proliferation and differentiation by shortening the cell division cycle (Liu et al., 2008). In addition, Si is closely related to the metabolism of bone cartilage and has good therapeutic effects on osteoporosis, promotes syntheses of collagen and proteoglycans in bone cartilage, plays a special metabolic role in bone growth, and has positive impacts on bone growth, osteogenesis, cell differentiation and proliferation. The lack of Si has an important impact on growth, development and bone metabolism (Mohammadi and Sepantafar, 2016).

Preparation of antibacterial coatings on Ti surfaces through modification of those surfaces is also a current object of attention in this field. Some inorganic elements, such as copper, magnesium, fluorine and so on, have good antibacterial properties and are used for surface modifications of Ti (Zhou et al., 2018; Zhao et al., 2019; Zhang et al., 2022). Antibacterial coatings containing Ag/Ag ions have attracted extensive attention. As an antibacterial agent, the ability of Ag/Ag ions to kill and inhibit a variety of microorganisms has long been widely known, and Ag/Ag ions can even kill bacteria with drug resistance (Zhang et al., 2018). In addition, Ag/Ag ions have other advantages, such as stable bactericidal ability and low cytotoxicity. Although biomaterials containing Ag ions have been widely studied and used to prevent the formation of biofilms on the surfaces of materials, it is still challenging to control the dose of Ag ions and obtain long-term antibacterial effects.

In view of the good biological activities of Si and Ag, we used plasma oxidation in this study to prepare porous TiO_2_ coatings containing Si and Ag (TCP-SA) on the surfaces of Ti. The surface properties of different samples, including surface morphology, element composition and chemical state, roughness, adhesion, hydrophilic/hydrophobic properties and ion release, were evaluated in detail. On the basis of *in vitro* experiments, the biocompatibility of the coating and the bioactivity in promoting bone and antibacterial activity were evaluated. Additionally, the bone integration performance of the coating was verified with an *in vivo* implantation experiment. Finally, the osteogenic properties of Ti implants with TCP-SA were evaluated by *in vivo* experiments. Our study is designed to provide a new idea and method for improving the biocompatibilities and bioactivities of Ti implants.

## 2 Materials and methods

### 2.1 Preparation and surface characteristics of experimental samples

Using wire cutting technology, a Ti rod was processed into a disc with a diameter of 14.5 mm and a thickness of 2 mm, polished with sandpapers of different grits, and then washed with acetone, absolute ethanol and deionized water. Then, plasma oxidation was conducted after drying.

The plasma oxidation power supply was a microarc oxidation (MAO)-600-11a system, with a Ti sheet as the anode and stainless steel as the cathode. The plasma oxidation time was set to 5 min, the frequency was set to 1000 Hz, and the duty cycle was set to 30%. After plasma oxidation was completed, the samples were washed and dried.

The initial electrolyte solution comprised calcium acetate and calcium glycerophosphate dissolved in deionized water, pure Ti sheets were marked as Ti, Ti was marked as TCP after plasma oxidation in the electrolyte described above, and the sample oxidized after adding sodium silicate and Ag nitrate to the initial electrolyte solution was marked as TCP-SA. The composition of the electrolyte is shown in Table 1.

**TABLE 1:** Electrolyte components of different coatings.

Coating	Aqueous electrolyte concentration (M)			
	Calcium acetate	Calcium glycerophosphate	Sodium silicate	Silver nitrate
Ti	—	—	—	—
TCP	0.2	0.05	—	—
TCP-SA	0.2	0.05	0.06	0.03

The surface morphologies, structures, elemental compositions and valence states, surface roughnesses and hydrophilicities of different samples were analyzed by scanning electron microscopy (SEM), X-ray diffraction (XRD), X-ray photoelectron spectroscopy (XPS), a profiler and a contact angle meter (CA). A nanomechanical property testing system with a diamond indenter was used to test the bonding strength of the coating.

The samples were washed with deionized water, dried naturally, immersed in 100 ml of PBS buffer solution, and placed in a 37°C constant temperature incubator. All PBS solutions containing the measured ions were collected at 1, 2, 3, 4, 5 and 7 days, and the contents of silicon ions and silver ions released into the PBS were collected at various times and detected with inductively coupled plasma‒mass spectrometry (ICP‒MS). The dynamic release behavior was analyzed.

### 2.2 Cell culture

MG63 cells in α-MEM medium were used in this study, and the cells were incubated in a 37°C cell incubator containing 5% CO_2_. When the cell confluence reached 80%, the cells were digested with 0.25% trypsin and subcultured at a ratio of 1:3. The third generation was used for *in vitro* experiments.

### 2.3 Cell adhesion and extension

A total of 2×10^4^ cells/well were inoculated on the surface of each group of samples and cultured in a 37°C, 5% CO_2_ constant temperature incubator, and the culture was terminated 72 h later. The samples were fixed with glutaraldehyde and osmic acid in turn, dehydrated with gradient alcohol, replaced with isoamyl acetate, dried at the critical point, and sprayed with gold on the surfaces of the samples, and the morphology of osteoblast adhesion and extension was observed by FE-SEM.

### 2.4 ECM mineralization

In this study, alizarin staining was used to evaluate the mineralization level of the extracellular matrix on the surfaces of different samples. The cell inoculation density and culture method used were the same as above. The culture was terminated on the 14th day after inoculation, glutaraldehyde was fixed, alizarin was stained, and extracellular mineralization was observed under a stereomicroscope.

### 2.5 *In vitro* antibacterial properties

#### 2.5.1 Recovery and culture of bacteria

To evaluate the antibacterial performance of the sample, the Gram-positive bacterium *Staphylococcus aureus* was selected for antibacterial performance evaluation in this study. The bacteria were transferred to flat solid medium and cultured at 37°C for 24 h. The activated bacteria were inoculated into liquid medium and cultured overnight at 37°C. A bacterial solution concentration of 1.0 × 10^5^ cfu/ml diluent was used as the bacterial solution for the experiment.

#### 2.5.2 Fluorescent staining

Fluorescent staining was used to evaluate the survival and death rates of bacteria on the surface of the sample. The bacteria were cultured on the surface of the sample for 24 h, and 50 μl of the acridine orange (AO)/propidium iodide (PI) 1:1 mixed dye was dripped evenly on the surface of the sample and dyeing was performed away from light. Bacterial survival was observed under an inverted fluorescence microscope.

### 2.6 Animal *in vivo* research

#### 2.6.1 Preparation of animal model

Studies involving animals were reviewed and approved by the Ethics Committees of Guizhou Provincial People’s Hospital. Twelve healthy 10- to 12-week-old male SD rats weighing 300–350 g were selected. All rats were purchased from the animal center of Sichuan University. The samples were divided into an experimental group (TCP-SA-coated Ti) and a control group (Ti). The samples were processed into cylinders with a diameter of 3 mm and a length of 5 mm by wire cutting. After the samples were disinfected, the experimental group and the control group were implanted into the bilateral femoral condyles of rats at the same time. The specific method was as follows. Pentobarbital sodium anesthesia was used. A 2 cm longitudinal incision was made in the lateral femoral condyle, the lateral femoral condyle was separated and exposed, and the bone defect model with a diameter of 2.6 mm was prepared by drilling perpendicular to the bone surface with the help of a surgical electric drill. The lateral femoral condyles of the experimental group and the control group were implanted at the same time, the wounds were sutured and bandaged, and penicillin was injected intramuscularly for anti-infection treatment after the operation.

#### 2.6.2 Osteogenesis performance

Four weeks after the operation, the rats were killed, and the femoral condyles of the rats in the experimental group and the control group were cut. The surfaces of the implants were observed for necrosis, infection, fiber wrapping and new bone tissue, and then micro-CT and histological evaluations were performed. For micro-CT detection, all samples were fixed with alcohol, and micro-CT scanning was used for three-dimensional reconstruction. Through the built-in software of the micro-CT system, the region of interest was set, and the bone volume fraction (bone volume/total volume, BV/TV%) was measured. For histological evaluation, the specimens after micro-CT scanning were dehydrated and embedded after alcohol fixation, hard tissue sections were stained with toluidine blue and acid fuchsin, and Image-Pro Plus 6.0 image analysis software was used for semiquantitative analyses of new bone tissue.

### 2.7 Statistical analyses

All data are expressed as the mean ± standard deviation and were analyzed by SPSS 18.0 software. One-way ANOVA and the SNK test were used to compare the differences between groups. *p* < 0.05 and *p* < 0.01 indicate statistical significance.

## 3 Results

### 3.1 Surface morphologies, phases and elemental compositions of sample groups

**FIGURE 1:**
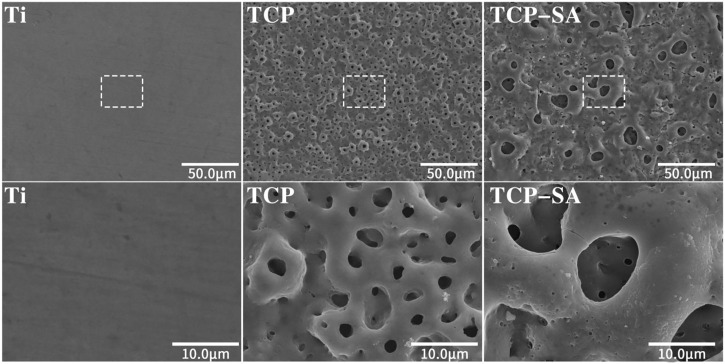
SEM surface morphology of different samples under low and high magnification (×600 and ×3500). The plasma oxidation time is 5 min, and the reaction temperature is room temperature.

Figure 1 shows the SEM surface morphologies of different samples. The Ti group differed significantly from the other groups, and the surfaces were smooth with sandpaper grinding marks. The TCP and TCP-SA samples were similar, and there were no obvious differences. At low magnification, the original morphology of Ti no longer existed, and the surface was covered by micropores of different sizes. Under magnification, the surfaces of various coatings were rough, the micropores were irregular ellipses, and there were connections between different micropores. In addition, there were a small number of ablated particles on the surfaces of the TCP-SA samples.

**FIGURE 2:**
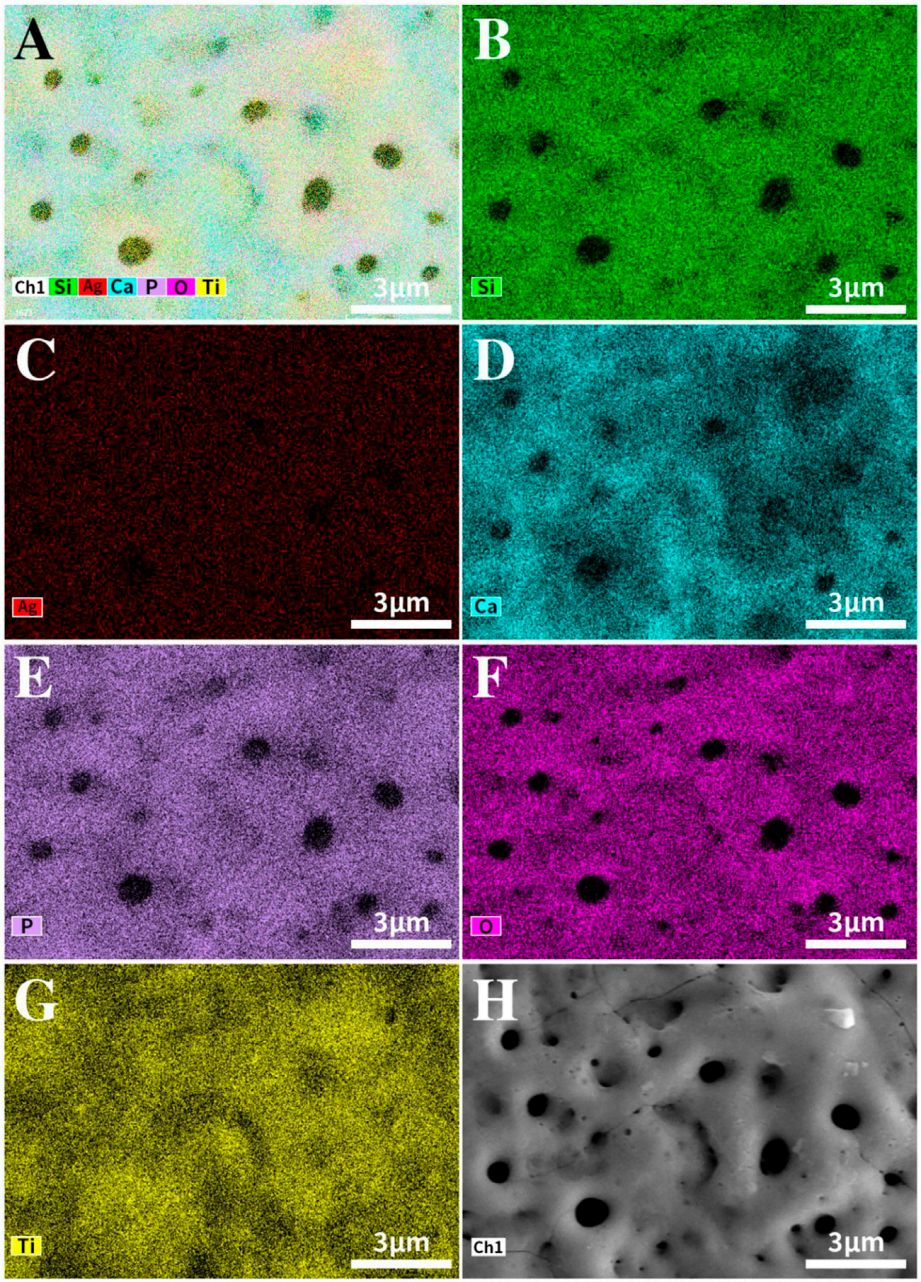
EDS mapping image of the TCP-SA sample. **(A)** Composite map of TCP-SA, **(B)** Si, **(C)** Ag, **(D)** Ca, **(E)** P, **(F)** O, **(G)** Ti and **(H)** surface morphology.

Figure 2 shows the EDS mapping images of TCP-SA. Different colors represent different elements. As seen in Figure 2A, the surface of TCP-SA comprised Ti, Ca, P, O, Si and Ag, among which Ca, P, Si and Ag were all from the electrolyte solution. The mapping diagram showed that all elements in the coating were evenly distributed on the surfaces and holes of the coating.

**FIGURE 3:**
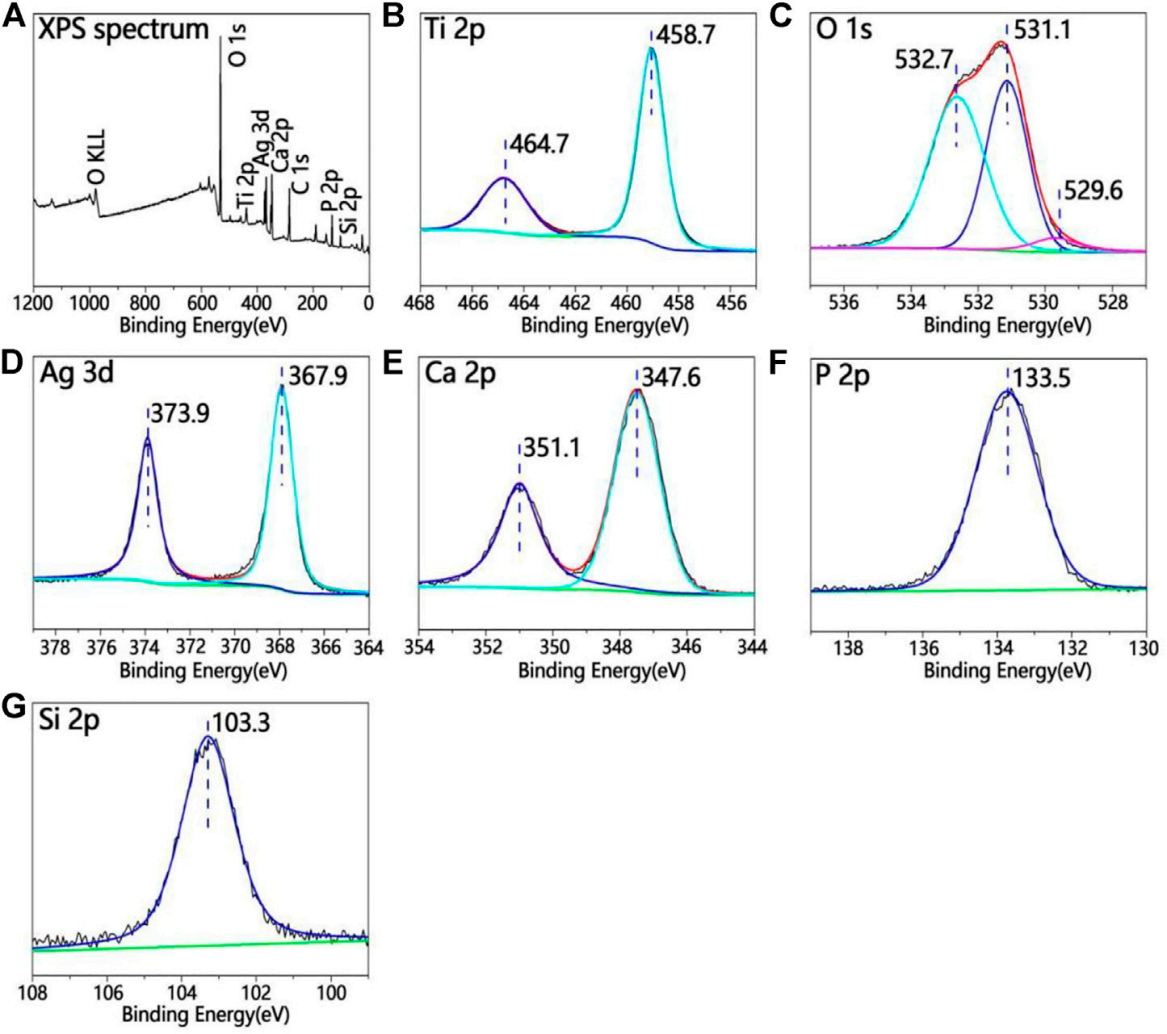
**(A)** XPS full spectrum for TCP-SA and **(B)** Ti 2p, **(C)** O 1s, **(D)** Ag 3d, **(E)** Ca 2p, **(F)** P 2p and **(G)** Si 2p XPS data.

Figure 3 shows the XPS full spectrum of the TCP-SA. Figure 3A shows the full X-ray photoelectron spectrum for the TCP-SA. The TCP-SA comprises Ca, P, O, Si and Ag. The peak in the Ti 2p spectrum corresponded to porous TiO_2_. The Ca 2p peaks were situated at 351.1 eV and 347.6 eV, and the P 2p peak was situated at 133.5 eV, which showed that the Ca 2p and P 2p binding energies resulted from Ca-phosphate phases. The Si 2p peak was located at 103.3 eV, which was assigned to the Si 2p binding energy of SiO_2_. The Ag 3d spectrum had two peaks (373.9 eV and 367.9 eV), which corresponded to Ag_2_O. The O 1s spectrum had three peaks: the peak located at 532.7 eV was assigned to the O 1s binding energy for SiO_2_, the other peak at 531.1 eV corresponded to Ca_3_(PO_4_)_2_, and another peak located at 529.6 eV was assigned to Ag_2_O.

**FIGURE 4:**
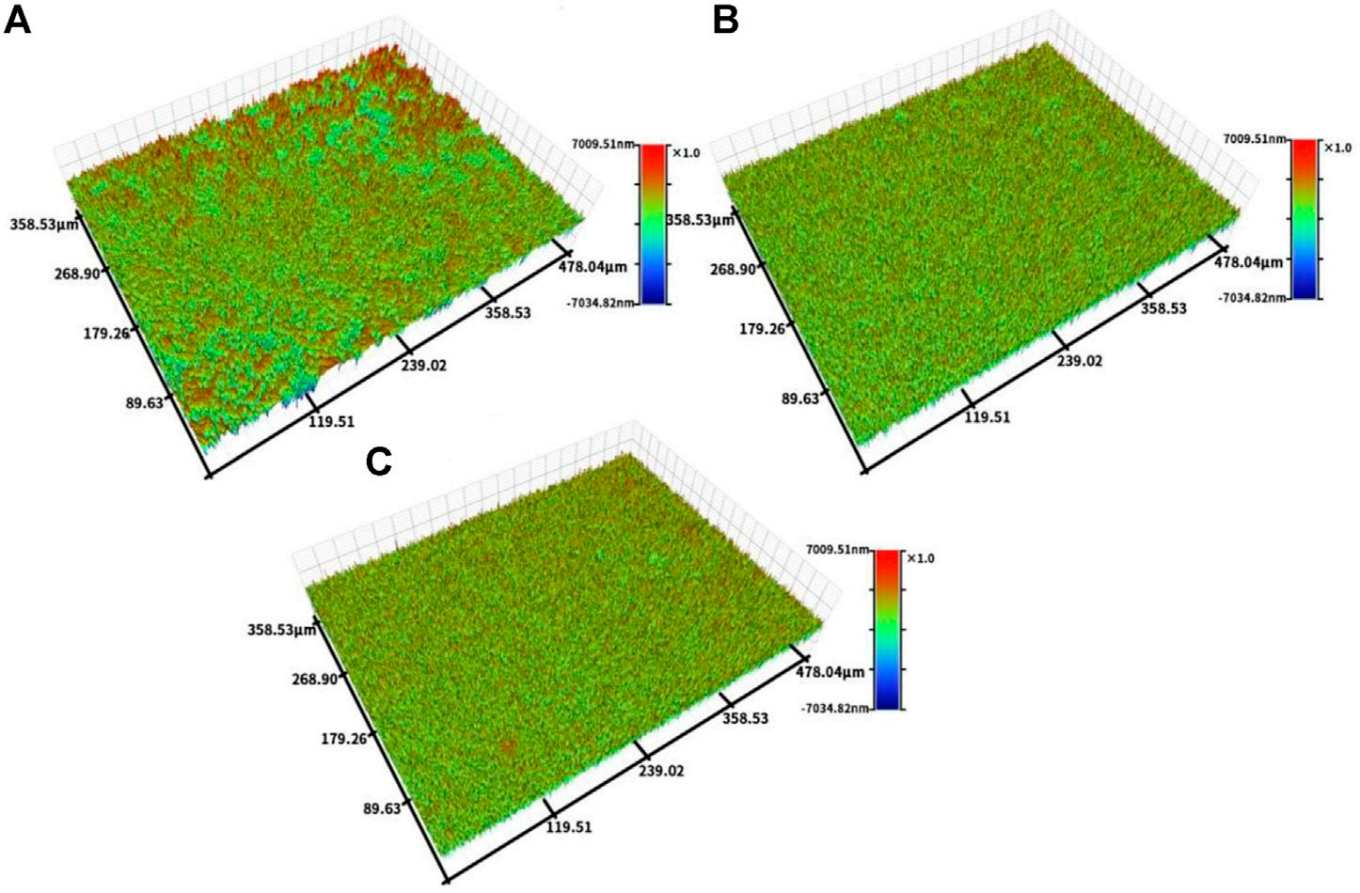
Profiler surface topographies for each group of samples, and **(A)** Ti, **(B)** TCP and **(C)** TCP-SA.

Figure 4 shows the profiler surface topographies determined for each group of samples. The surface roughness distributions for each group of samples were uniform, and the TCP and TCP-SA samples showed volcano-like multilevel pore-void structures. Further quantitative analyses of the surface roughness values showed a Ti Ra of 626.69 ± 10.29, a TCP Ra of 939.33 ± 20.28 and a TCP-SA Ra of 1152.55 ± 67.65. The roughness for each group of samples conformed to this trend and decreased in the order TCP-SA > TCP > Ti. TCP-SA increased the surface roughness of the Ti after plasma oxidation.

**FIGURE 5:**
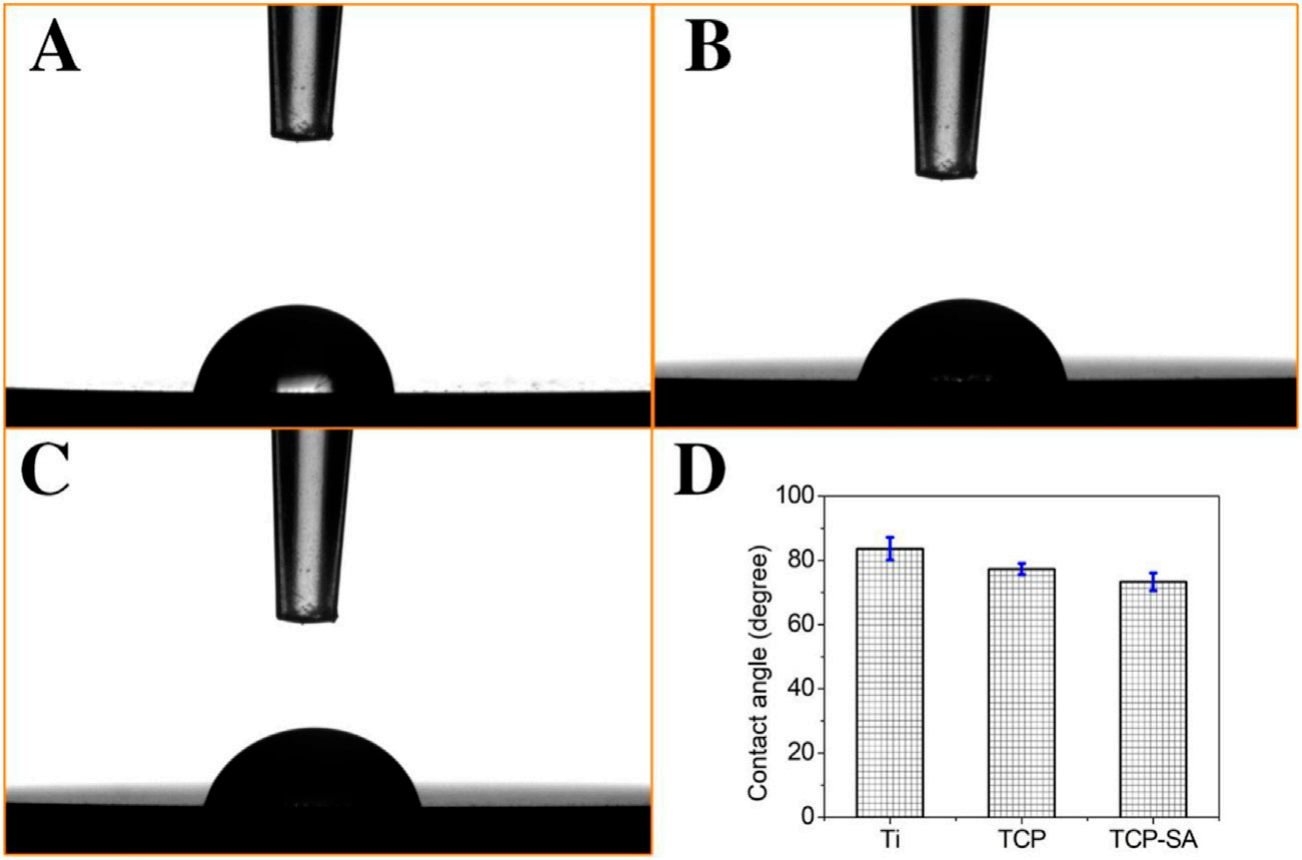
Static contact angles for each group of samples, and **(A)** Ti, **(B)** TCP, **(C)** TCP-SA and **(D)** bar chart of contact angle.

Figure 5 shows the static contact angles for each group of samples. In this study, the hydrophilicity of each group of materials was observed through the contact angle. The hydrophilicity is closely related to the microstructure of the surface of the material, and the wettability of the surface can reflect the surface energy of the material. In this study, the contact angles of each group of materials are ranked as follows: Ti > TCP > TCP-SA.

**FIGURE 6:**
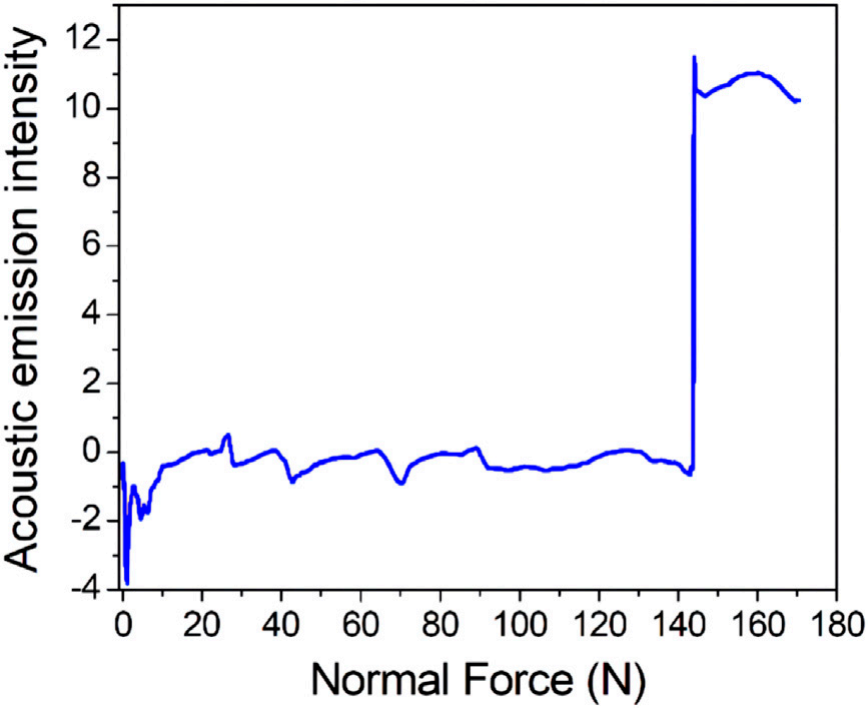
Acoustic emission spectrum for the coating (the initial load was 0 N, the final load was 150 N, the scratch speed was 2 mm/min, the loading rate was 50 N/min and *n* = 3).

Figure 6 shows the acoustic emission spectrum of the coating. In this study, a nanomechanical property testing system equipped with a diamond indenter was used to test the bonding strength of the coating and titanium. The results showed that adhesion between the coating and titanium matrix was greater than 143.0 ± 3.6 N. The coating had a high bonding strength with the titanium substrate.

**FIGURE 7:**
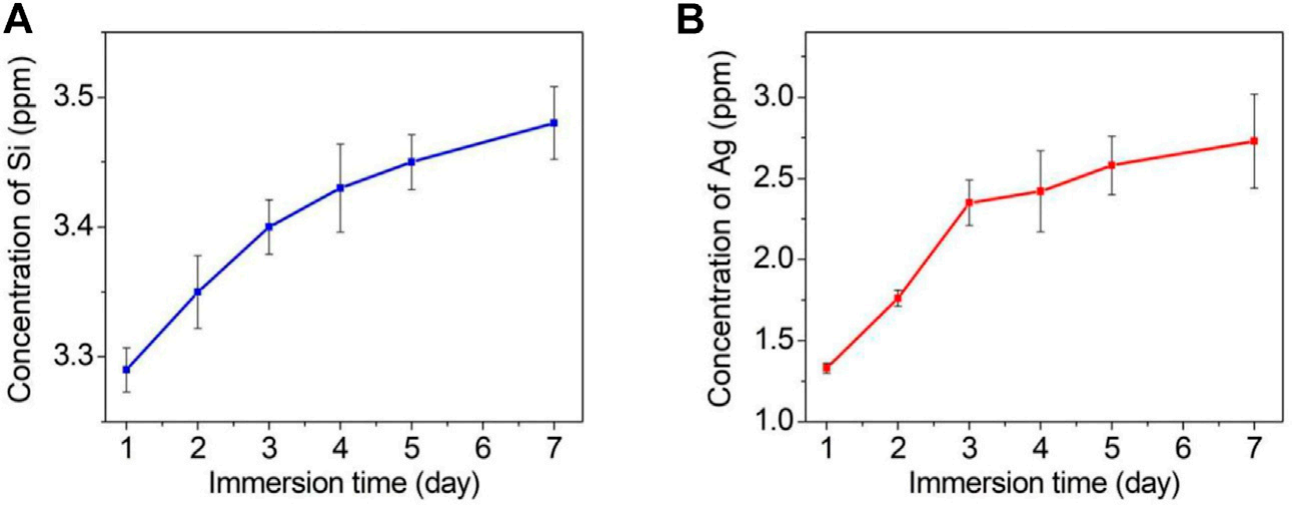
Cumulative release profiles for silicon ions **(A)** and silver ions **(B)** from the TCP-SA after immersion in PBS of different durations.

Figure 7 shows the cumulative amounts of released silicon (a) and silver (b) detected by ICP‒MS after the TCP-SA was immersed in PBS solution for 1 day, 2 days, 3 days, 4 days, 5 days and 7 days. It is obvious that the total contents of silicon ions and silver ions released from the coating gradually increased with prolonged immersion time, but the ion release rate was fast at the beginning of immersion, and then the ion release rate gradually slowed. The contents of silicon ions and silver ions in the pores inside the coating were higher than those on the outer surface of the coating, thus ensuring long-term stable release of ions. The release of long-acting and safely controllable silicon and silver ions also provided good support for subsequent osteoblast-related experiments and antibacterial experiments.

### 3.2 Cell adhesion and spreading on different sample surfaces

**FIGURE 8:**
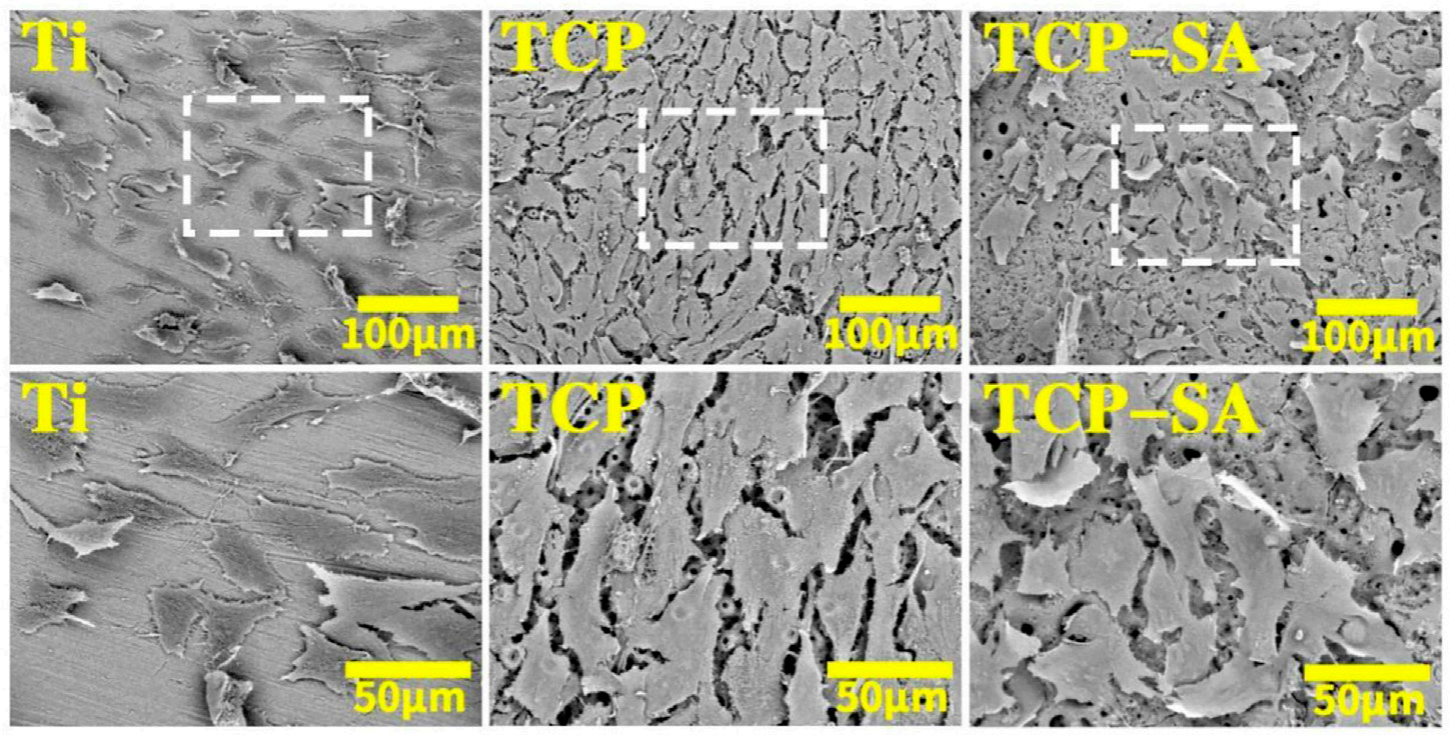
SEM results showing cells spread on different sample surfaces.

Figure 8 shows FE-SEM images of osteoblast adhesion and extensions on different sample surfaces. In the Ti group, sporadic cells were seen on the surface, the number of cells was small, and there were few cell surface protrusions. The osteocytes comprising TCP were densely packed and exhibited polygonal shapes, relatively plump cells, and abundant particles on the surface. Similar to the cells in the TCP group, the surface cells of the TCP-SA group were polygonal and regularly arranged, with local cell accumulation and multilayered growth.

### 3.3 ECM mineralization

**FIGURE 9:**
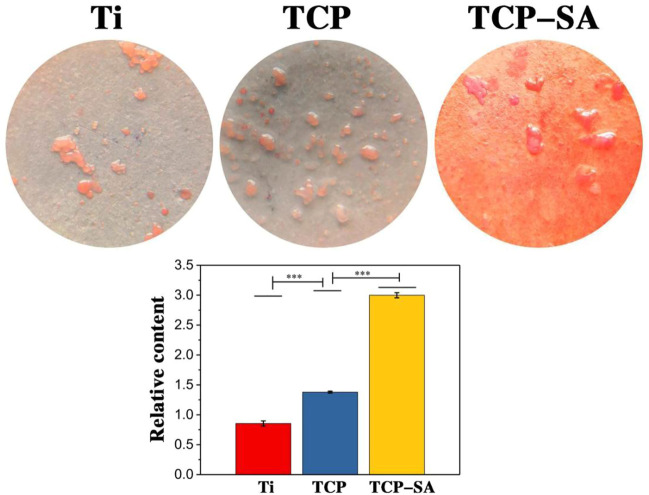
Results of extracellular matrix mineralization on the surfaces of different samples. Data were expressed as mean ± SD (*n* = 3). ***p < 0.01.

Figure 9 shows the results of extracellular matrix mineralization on the surfaces of different samples. We can see that the staining depths of different samples were different. The deeper the staining, the higher the degree of mineralization. The degree of mineralization for each group of samples decreased in the order TCP-SA > TCP > Ti. Furthermore, the degree of mineralization for TCP-SA was higher than that of the TCP composite with Ti rent, and the difference was significant.

### 3.4 *In vitro* antibacterial experiments

**FIGURE 10:**
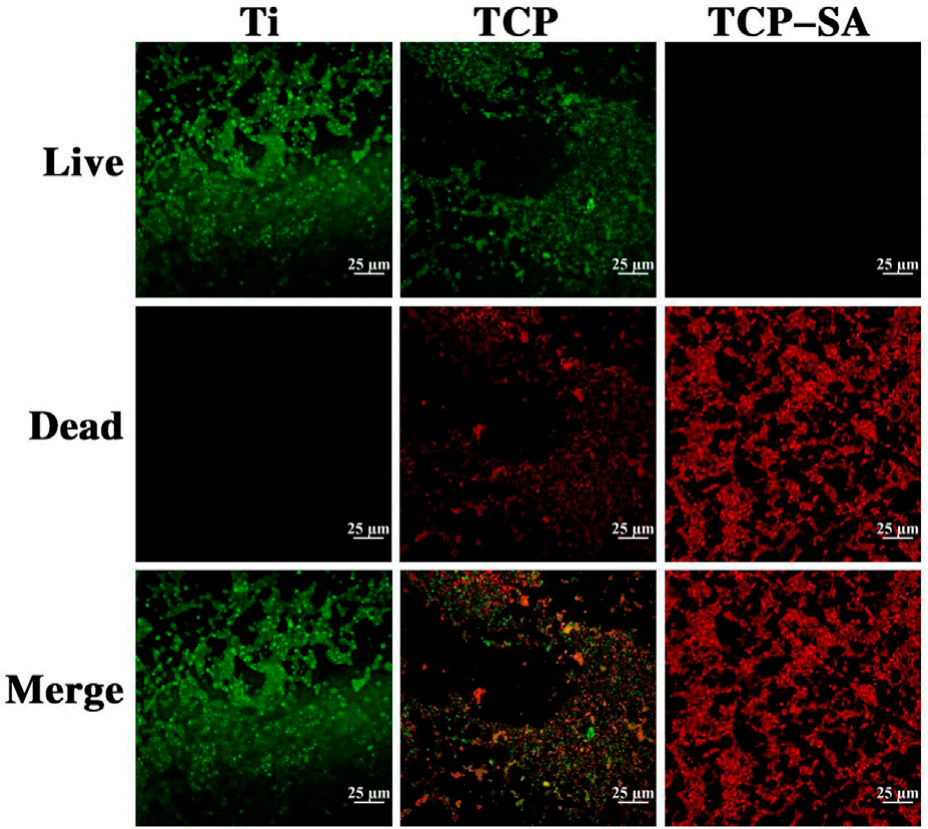
Fluorescence staining results for live and dead bacteria in different samples.

Figure 10 shows the fluorescent staining results with live and dead bacteria for different samples. As acridine orange (AO) binds to DNA in living cells with intact cell membranes, it emits green fluorescence. Propidium iodide (PI) enters the interiors of necrotic bacteria, binds to its DNA, and emits red fluorescence. By observing the colors of the bacteria, the survival rate for bacteria on the coating surface is reflected. Obviously, the bacteria on the Ti surface were all green live bacteria, indicating that Ti has poor antibacterial activity. A large number of live bacteria were still visible on the surface of the TCP group, but the number was significantly less than that of the Ti group. The live bacteria adhering to the surfaces of the TCP-SA samples had almost disappeared, and they were all red (dead) bacteria. This shows that the porous Si/Ag-TiO_2_ coating had good antibacterial properties.

### 3.5 Gross observations in animals

The surfaces of the implants in both groups were wrapped by tissue, the TCP-SA group had osseous connections with the surrounding bone tissue, and there was little bone trabecular formation. The Ti group was mainly surrounded by fibrous tissue. No necrosis or infection was found in any of the samples.

### 3.6 Micro-CT results

**FIGURE 11:**
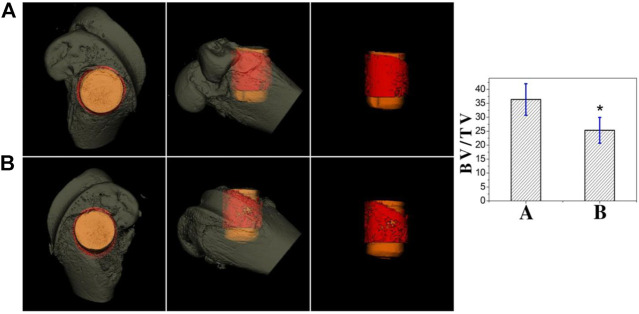
Micro-CT results of different samples at 4 weeks after implantation (**(A)**: TCP-SA group; **(B)**: Ti group). Data were expressed as mean ± SD (*n* = 3). *p < 0.05.

Figure 11 shows the micro-CT results for two sets of samples. The implants in both groups were surrounded by new bone. Compared with the Ti group, the new bone tissue in the TCP-SA group was significantly increased, the bone tissue was denser, and no obvious osteonecrosis was found. The bone volume/total volume (BV/TV) ratio represents the total amount of bone formation. Through quantitative analysis, the BV/TV in the TCP-SA group was higher than that in the Ti group and the difference was statistically significant (*p* < 0.05), indicating that the porous Si/Ag-TiO_2_ coating on the Ti surface promoted implantation osseointegration.

### 3.7 Histological staining of animal specimens

**FIGURE 12:**
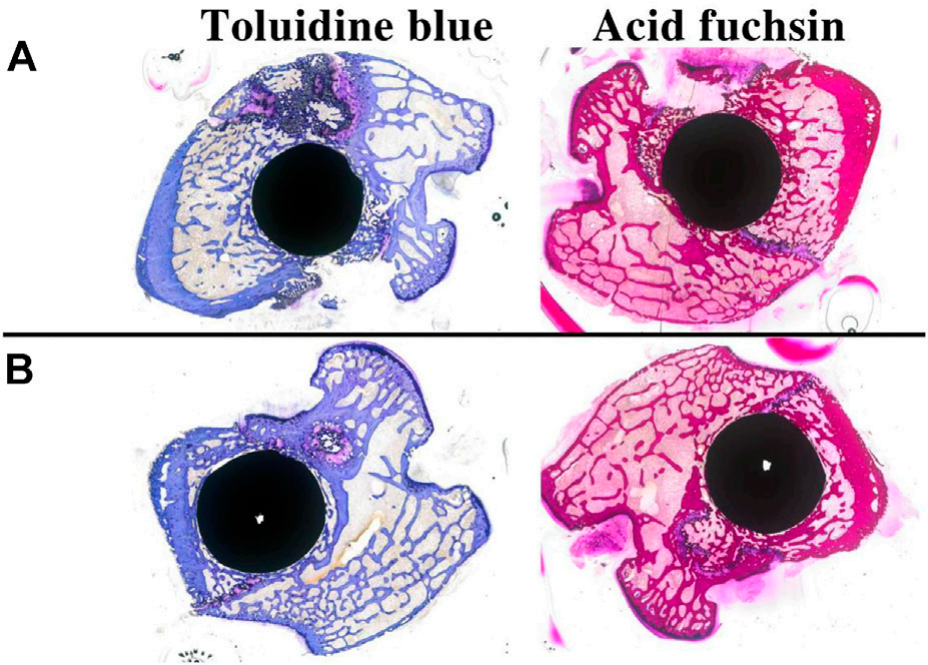
Toluidine blue and acid fuchsin staining results for different samples at 4 weeks after implantation (**(A)**: TCP-SA group; **(B)**: Ti group).

Figure 12 shows the results of toluidine blue and acid fuchsin staining for two sets of samples. As with the micro-CT results, and compared with the Ti group, the porous Si/Ag-TiO_2_ coating peri-implant group had more new bone tissue, and the trabecular bone was denser. The new bone area ratio was used to quantify the new bone tissue on the surface of different implants. Software analysis showed that the area ratio of new bone tissue in the experimental group (33.58 ± 5.01) was higher than that in the control group (24.89 ± 6.15), and the difference was statistically significant (*p* < 0.05). The toluidine blue and acid fuchsin staining results were consistent with the micro-CT results.

## 4 Discussion

Orthopedic biomaterials with both osteogenic and antibacterial properties are urgently needed. To improve the success rate of osseointegration for bone tissues surrounding Ti implants and reduce the incidence of Ti implant infections, the common method is to modify the surfaces of the Ti implants. Through surface modification, the surface morphologies, surface chemical compositions and surface roughnesses of Ti implants can be changed, and the biocompatibilities and bioactivities of the Ti implants can be improved (Zhang et al., 2020; Zhang T. et al., 2021). In this study, the coating prepared by plasma oxidation technology had a porous structure, which increased the roughness of the material. More importantly, because Si has good cellular activity and cytocompatibility and Ag has strong antibacterial activity, we introduced the bioactive Si and Ag into the Ti surface through plasma oxidation technology and improved the osteogenesis and antibacterial properties of the Ti alloy *via* slow release of Si and Ag.

Doping bioactive elements into the surfaces of Ti implants to improve the bioactivities and biocompatibilities of Ti implants is currently a major focus of researchers. Plasma oxidation has been described in the literature as one of the basic methods of Ti surface modification. Introduction of zinc, magnesium, copper and other elements into the surfaces of Ti implants through plasma oxidation significantly improves the biological activities of Ti implants and promotes bone integration between Ti implants and surrounding bone tissues (Zhou and Zhao, 2016; Zhao et al., 2020b; Lv et al., 2021). In this study, porous TiO_2_ coatings doped with Si and Ag were prepared on the surface of medical Ti by adding sodium silicate and silver nitrate with different qualities to the basic electrolyte solution for plasma oxidation. Successful incorporation of Si and Ag into the TiO_2_ coating was confirmed by EDS mapping and XPS. Si has been proven to have good osteoinductive properties *in vitro*, and Si-containing biomaterials have also been used in the clinic. Ag has good antibacterial properties, and nano Ag patches used in the clinic have shown good antibacterial properties. Based on the bone-promoting properties of Si and the good antibacterial properties of Ag, the two elements were introduced into a Ti plasma oxidation coating with the help of plasma oxidation. There is no similar report in the literature.

The bonding strength between the surface coating and the matrix of an orthopedic implant is required for the internal stability of the implant and is also the key to success for implant implantation. It is very important to prevent complications such as implant loosening and sinking. Different coating materials, different coating preparation methods and different substrates affect the bonding strengths of coatings and substrates. In this study, we tested the bonding force between the coating and the sample with a nanomechanical property testing system equipped with a diamond indenter. The results showed that when the load reached 143.0 ± 2.6 N, the acoustic emission signal suddenly increased, indicating that the coating failed under this pressure; this confirmed that the coating and titanium had good bonding strength that would meet the needs of orthopedic implants. The long-term, safe and controllable release of silicon and silver ions enables the implantation to promote bone formation and antibacterial activity. In this study, we soaked the porous samples in PBS solution and observed their release patterns. It was found that silicon and silver ions were released gradually over time. Moreover, since the porous coating also contained large numbers of silicon and silver ions, long-term and slow release of silicon ions and silver ions was ensured, providing support for the bone-promoting and antibacterial properties of the implants *in vivo*.

The surface roughnesses of Ti implants have different biological effects on cells and tissues, especially for cell adhesion and expression of extracellular matrix proteins, which are affected by the surface micromorphology. Compared with a smooth surface, a rough surface is more conducive to differentiation of osteoblasts. Studies have confirmed that osteoblasts more easily adhered and proliferated on rough and porous Ti surfaces (Kulkarni et al., 2015). Only by adhering more cells to the Ti surface and making the morphology of the Ti surface more suitable for adhesion and proliferation of bone cells can we better promote integration between the implant and the surrounding bone tissue. Therefore, turning the smooth surface into a rough surface through surface modification technology is conducive to adhesion and growth of cells and to early stability of the implant. In addition, this study showed that the alkaline phosphatase activities of cells were higher on the rough implant surface, which caused more cells to adhere to the implant surface and improved bone integration (Minagar et al., 2013). The porous Si/Ag-TiO_2_ coating prepared in this study by plasma oxidation showed that the surface roughness was significantly higher than that of pure Ti, which was conducive to osteogenic differentiation of osteoblasts.

After Ti implants are implanted *in vivo*, adhesion and extension of osteoblasts on the surfaces of the materials enable subsequent cell proliferation and differentiation. In this study, the adhesion and extension properties of osteoblasts on the surfaces of each group of materials were observed by scanning electron microscopy. The results showed that the cells on the surface of the porous Si/Ag-TiO_2_ coating were polygonal and arranged regularly, and cell accumulation occurred. The porous Si/Ag-TiO_2_ coating promoted adhesion and extension of osteoblasts. We speculate that the porous Si/Ag-TiO_2_ coating promotes adhesion and extension of the osteoblasts, which is related to the porous morphology and the doping with Si. As a necessary trace element for the human body, Si is mainly concentrated in areas where bone development is active (such as areas where osteoblasts are active) and plays an important role in bone formation. Studies have shown that silicon-containing materials improved the surface biological activities of materials by releasing silicon ions. The released silicon ions combined with the OH groups in the surrounding environment to form Si-OH groups, and the Si-OH groups induced nucleation of apatite. At the same time, calcium ions and P ions in the surrounding environment were gathered to form apatite. By upregulating the expression of genes and proteins related to osteoblastic differentiation, the osteoblasts can promote adhesion, proliferation and differentiation.

When extracellular matrix secretion matures, osteoblasts enter the extracellular matrix mineralization stage, which is an important stage in the process of bone formation. The results of this study showed that the amount of matrix mineralization in the porous Si/Ag-TiO_2_ coating group was significantly higher than those in the TCP and Ti groups, which we speculate is related to the surface properties of the coating and the release of elemental Si in the coating. Doping with Si upregulated the proteins related to extracellular matrix mineralization, such as BSP and OCN, thus promoting mineralization of the extracellular matrix.Our experimental results were very consistent with previous literature reports indicating that Si promotes proliferation, differentiation and and mineralization of osteoblast-related cells (He et al., 2018).

The antibacterial properties of the Ti implant surface provide another important index for measuring the biological activities of implants. After Ti implants are implanted into the body, a layer of protein is rapidly adsorbed on the Ti surface, which is conducive to cell adhesion as well as bacterial aggregation (Veerachamy et al., 2014). If bacteria are successfully colonized, they multiply and gather rapidly and finally form bacterial biofilms. The presence of this biofilm ensures that the internal bacteria will not be attacked by the host defense system or antibacterial agents through a variety of possible mechanisms, resulting in strong drug resistance. With expansion of the biofilm, inflammatory reactions appear around the implant, eventually leading to failure of the operation (Li et al., 2021; Li et al., 2022). Clinical research has shown that early prevention of bacterial biofilm formation is the most effective way to prevent inflammatory reactions around implants (Pavithra and Doble, 2008). Prevention of bacterial adhesion and biofilm formation with surgical implant materials has been widely studied.

As important antibacterial agents, Ag ions have several antibacterial characteristics. The first is slow-release contact reactions of Ag ions. Ag ions have high activities in liquids and are firmly adsorbed on the surfaces of bacteria, penetrate the bacterial cell membrane or cell wall, and enter the bacteria, thereby limiting bacterial activity. More importantly, when Ag ions kill bacteria, a series of reactions occurs in the bacteria, leading to dysfunction of the surrounding environment; this is manifested as respiratory interruption and metabolic cessation, finally leading to death of the bacteria (Tang and Zheng, 2018). After the bacteria die, Ag ions enter other bacteria and repeat the antibacterial reactions, which is one of the reasons for their long-term antibacterial properties (Yin et al., 2020). The second characteristic is a catalytic reaction: due to photocatalysis with Ag ions, oxygen and water in the air are activated. After negative oxygen ions and hydroxyl radicals act the bacteria together, the proliferation and metabolic processes of bacteria are blocked, and they eventually die.

In this study, we observed the antibacterial properties of each group of samples with scanning electron microscopy. The results showed that the porous Si/Ag-TiO_2_ coating group had strong antibacterial properties. Almost no living bacteria adhered to the surface of the porous Si/Ag-TiO_2_ coating, and the colonies adhering to the surface of the porous Si/Ag-TiO_2_ coating had died or were on the verge of death. In contrast, large numbers of bacteria adhered to the surfaces of Ti and TiO_2_ pores. Our research results are consistent with those reported in the literature. Shimabukuro et al. (2021) prepared Ti containing both Ag and Zn on the surface *via* two-step micro arc oxidation, and the results showed that MAO-treated samples containing Ag and Zn showed good antibacterial activity against *Escherichia coli*. Similarly, Song et al. (2009) formed a Ag-containing Ca-phosphate coating on a Ti substrate with MAO. *In vitro* research showed that the Ca-phosphate coating prepared with a low Ag concentration electrolyte had antibacterial activity *in vitro* but no cytotoxicity, indicating that biocompatible Ca-phosphate coatings with antibacterial activities could be adhered to Ti implants with MAO. Studies have shown that bacterial adhesion to the surfaces of implants is the first step in the formation of biofilms, and it is the only reversible stage in the formation of biofilms. Once biofilms are formed, they are extremely difficult to remove. Therefore, preventing bacterial adhesion in the initial stage is the most effective and promising method to prevent biofilm formation (Song et al., 2009).

This study further evaluated the osteogenic properties of Ti implants with porous Si/Ag-TiO_2_ coatings through *in vivo* implantation experiments. Micro-CT and histological staining are common techniques used for observing the osteogenic properties of *in vivo* implants. Micro-CT can be used to directly evaluate the osteogenic performance of an implant. In this study, we chose bone volume/total volume (BV/TV) as the detection index. BV/TV represents the total amount of bone formation and is an important indicator of implant osseointegration. Our results showed that the BV/TV of the porous Si/Ag-TiO_2_ coating group was higher than that of the Ti group. Using micro-CT, we further observed the bone tissue around the implant through hard tissue sections. The results of toluidine blue and acid fuchsin staining showed that formation of new bone in the porous Si/Ag-TiO_2_ coating group was greater than that in the Ti group, and the surrounding newly formed bone was in direct contact with the implant. The porous Si/Ag-TiO_2_ coating on the Ti surface promoted integration of the implant and the surrounding bone tissue. The results of the *in vivo* studies were consistent with previous *in vitro* research results. The porous structure of the Ti surface, appropriate roughness and the combined action of Si and Ag ions improved the osteogenic properties of Ti implants.

Although the porous Si/Ag-TiO2 coating prepared in this study stimulated cell response and indicated antibacterial properties, there are still shortcomings. We lack experimental research on the molecular mechanisms by which this coating promotes bone formation, which is necessary to improve and optimize the coating. In addition, the human body has a complex microenvironment, and we have not conducted *in vivo* studies confirming our *in vitro* findings.

In this study, Si and Ag were innovatively mixed into porous TiO_2_ coatings by the plasma oxidation method to prepare porous Si/Ag-TiO_2_ coatings, which showed good microporous surface morphologies and suitable roughness. In addition, the coating exhibited strong bonding with the titanium substrate. *In vitro* studies showed that the porous Si/Ag-TiO_2_ coating promoted adhesion and extension of osteoblasts, differentiation of osteoblasts and mineralization of the extracellular matrix. More importantly, the porous Si/Ag-TiO_2_ coating inhibited the adhesion and proliferation of *Staphylococcus aureu*s and showed good antibacterial properties. On this basis, and through *in vivo* experiments, we further showed that porous Si/Ag-TiO_2_ coatings on the surfaces of Ti implants can promote integration between Ti implants and the surrounding bone tissue and show good biological activity. This study provides a new idea and method for improving the biological activity of Ti, which has good clinical application value.

## Data Availability

The original contributions presented in the study are included in the article/Supplementary Material, further inquiries can be directed to the corresponding authors.
